# Drug repurposing and phenotypic screening: innovative strategies for treating ultra-rare disorders

**DOI:** 10.3389/fmed.2024.1489094

**Published:** 2024-10-23

**Authors:** Adrien Paquot, Benoit Deprez, Terence Beghyn

**Affiliations:** ^1^APTEEUS, Lille, France; ^2^Univ. Lille, Inserm, Institut Pasteur de Lille, U1177 - Drugs and Molecules for Living Systems, EGID, Lille, France

**Keywords:** rare diseases, screening, translatability, phenotypic, drug discovery, repurposing, repositioning, inherited metabolic disorder

## Introduction

The pursuit of effective treatments for ultra-rare disorders presents a major challenge. Those conditions, characterized by their very low prevalence, genetic diversity, poor pathophysiological knowledge and diverse clinical manifestations, pose significant hurdles for traditional drug discovery and development approaches. However, amidst this complexity lies a promising avenue: drug repurposing, a strategy that involves identifying new therapeutic applications for existing drugs, trading uncertainty on disease knowledge for a better information on the candidate drug.

In recent years, the concept of drug repurposing has gained interest as a compelling strategy to expedite identification of therapeutic interventions for ultra-rare disorders. Unlike the development of new chemical entities, drug repurposing takes advantage of the wealth of existing pharmacological agents and their known safety and pharmacokinetics profiles. It offers a cost-effective and time-efficient alternative for addressing unmet medical needs. While academic research often supports this approach, the pharmaceutical industry tends to view it as a suboptimal investment, mostly because payers are hesitant to assign favorable prices, even when these medicines prove effective. In any case, the challenge for addressing an ultra-rare disease remains in a sufficient level of accessible knowledge on the drug and on the disease in order to derisk the development in the new indication.

**Figure 1:**
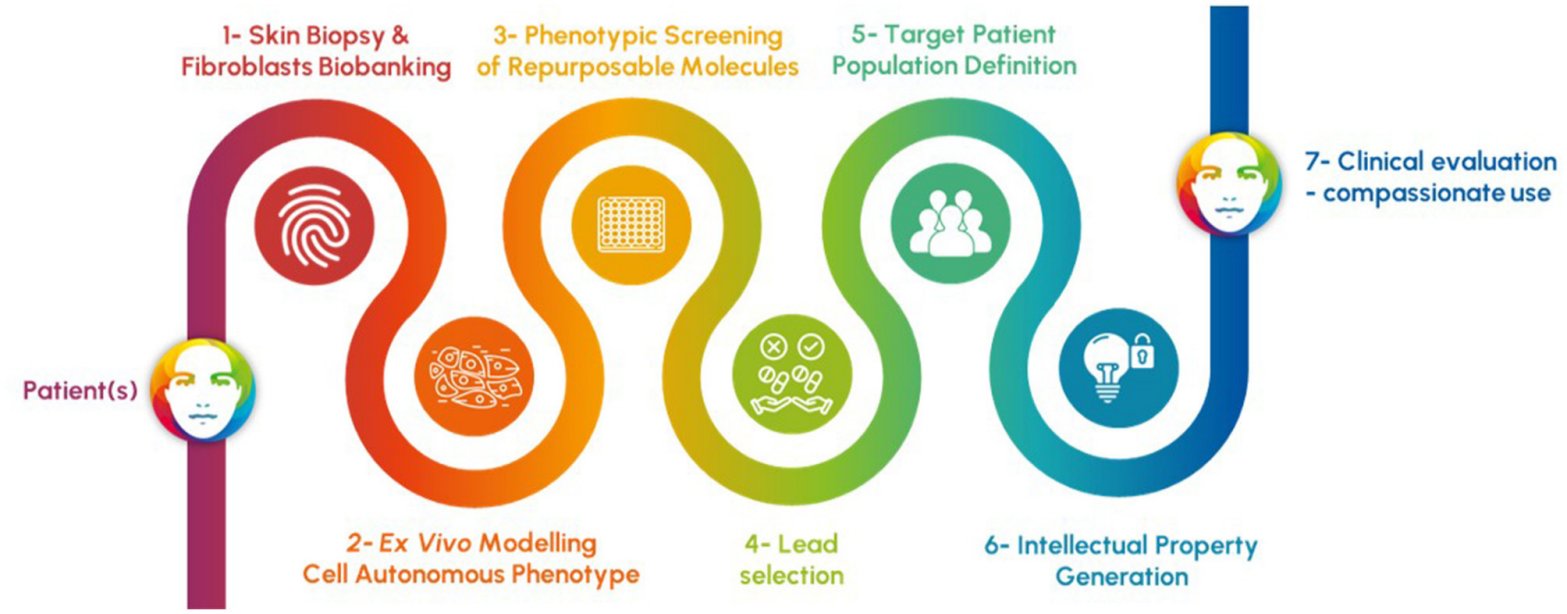
Process of rapid discovery engaging patients and relying on 1- the convenient access and use of skin fibroblasts, 2- the faithful ex vivo modeling of the causal defect of the patient symptoms in those cells, 3- a phenotypic screening of repurposable molecules based on the most relevant readouts, 4- the selection of best candidates to correct the cell-autonomous phenotype, 5- the definition of the responding population of patients through new testing of lead molecules, 6- the protection of the new use of drug candidates, and finally 7- the clinical evaluation through clinical trials or compassionate prescription procedures.

Apteeus has pioneered this field in systematically combining phenotypic screening and drug repurposing to identify new therapeutic opportunities for ultra-rare patients (Figure 1). Unlike target-based approaches, which focuses on specific molecular pathways or targets, phenotypic screening evaluates drug candidates based on their observable effects on cellular or organismal phenotypes. This holistic approach not only enables the discovery of novel therapeutic activities but also provides insights into the underlying disease mechanisms. It is particularly adapted to a category of disorders affecting cell-autonomous metabolism: the inherited metabolic disorders. Using primary patient cells as models and focusing on cellular phenotypes explaining the symptoms of the patients remains the most direct, reliable, and relevant strategy of drug discovery, especially when the defective gene is not connected to any known drug. In contrast with development initiated by clinical observations connecting the gene and an available candidate drug, this strategy starts with unbiased screening in the lab in such a way that the results obtained enable a direct translation to the clinic.

In this article, we explore the field of drug repurposing for addressing ultra-rare disorders, with a particular emphasis on the pivotal role of phenotypic screening. We endeavor to catalyze the development of much-needed therapies for individuals suffering from ultra-rare disorders.

## Drug repurposing for ultra-rare diseases

The pharmaceutical R&D process includes phases aiming at deepening knowledge of candidate drugs (1). The objective is to ultimately decrease the risk of efficacy or safety-related failures (2). Optimization of one property may compromise others (3), contributing to the complexity and duration of the drug R&D process. Typically, it takes 10 to 15 years to bring a new drug to market, with development costs proportional to the number and complexity of required steps (4, 5). High costs for developing a drug starts to pose challenges when targeting small patient populations, unless it is offset by a high selling price. This is evidenced by the unprecedented prices of several orphan drugs (6, 7), including the recent case of Zokinvy^®^, an orphan drug that treats Hutchinson-Gilford progeria syndrome sold at an astronomical cost: 1 million dollars per year.

In the efforts to address those cost issues in the field of rare diseases, the idea of drug repositioning was obvious. This strategy, focusing on compounds that have already undergone safety assessments, can significantly reduce drug development time, mitigate safety-related failure risks and minimize additional investments required for guarantying efficacy in a new indication (8).

The rationale of using an existing drug for a new indication can rely on two main scientific bases (9): (1) the discovery that some diseases share common biological targets and/or pathways, and (2) the concept of pleiotropic drugs.

The first concept involves connecting shared physio-pathological mechanisms. The low prevalence of a disease can unfortunately hinder research efforts to characterize it at the molecular level. When a connection is done, drug repurposing is an efficient way to answer the medical need reconciling research and development timelines with medical ones. A good illustration is the repurposing of alpelisib in Cloves syndrome (10). This specific inhibitor of PI_3_ kinase was successfully used to cure patients after the discovery that the Cloves syndrome was linked to a gain-of-function mutation in the corresponding gene of this kinase. It is however important to precise that unlike gain-of-function disorders, where inhibiting the mutated protein can restore normal function, addressing loss-of-function diseases is more challenging, as the target protein may no longer be present, or in a form that is not directly rescuable. In this case, pathway-wide or unbiased phenotypic screen is the only option to attempt to restore normal cell homeostasis.

The second concept relies on the polypharmacology of a small molecule. Every molecule, even the most optimized ones, are not so specific (11) and can own unsuspected properties in the context of illness. Thalidomide is a glaring example of polypharmacology (12). It was developed in the 1950s as a sedative for pregnant women, subsequently used as a therapy for leprosy and approved by the FDA in 2006 for treatment of multiple myeloma.

Usually, the potential of polypharmacology is revealed by clinical observations. Here again, the number of patients suffering rare diseases reduces the probability of observing such unsuspected effects. With the aim to disclose such favorable activities, phenotypic screening takes on its full meaning.

## Phenotypic screening

Although it has been somewhat neglected since the advent of target-based drug discovery (13), phenotypic drug discovery remains a potent strategy for assessing a drug's impact on disease models. Phenotypic drug discovery involves screening and hit selection steps based on measurable phenotypic outcomes from cell-based assays or model organisms, without prior knowledge of the specific drug target. The main advantage of this approach is to enable the identification of active compounds within complex biological systems that maintain intact signaling pathways, thereby better modeling distinct disease states (14).

Phenotypic strategies become particularly pertinent when no attractive target is known to address the causative defect of a disorder, a scenario often encountered in the context of rare diseases due to limited knowledge (8). Notably, a significant portion of pioneering medicines were discovered through phenotypic approaches that did not require a precise understanding of the molecular mechanism of action.

Associated with the objective of repurposing a drug, phenotypic screening facilitates a blind and unbiased exploration of the pharmacology of existing drugs. This approach promotes the identification of new, unsuspected activities and unprecedented targets for existing molecules. In 2020, Vincent et al. (15), published a methodology for developing phenotypic screens. The most important parameter is the relevance of the assay in relation to the disorder of the patient. It is essential to capture the largest biological space and increase confidence in the translation of the activity observed in the phenotypic assay and pathophysiological mechanisms identified in the patients.

To develop phenotypic drug discovery assays while maintaining a robust translatability to patients, Vincent et al. (16) proposed a set of guiding principles: the “3-rules.” (1) The biological system used in the phenotypic assay should exhibit a clear connection to the disease, such as patient-derived primary cells. It aims to replicate a relevant mechanism by which the disease is manifesting. As example, we performed research programs on several peroxisomopathies. Peroxisomes are cellular organelles, ubiquitously present in all cells. Even if a peroxisome deficiency affects mainly the liver and the central nervous system, skin fibroblasts, easily accessible and manipulable cells, also present a cell-autonomous phenotype. (2) The second rule warns about the use of artificial stimulus to induce the disease phenotype that can prevent the identification of relevant disease-modifying compounds. Therefore, systems not reliant on exogenous stimuli to induce the biological phenotype are preferred. (3) Finally, the readout of the assay should closely mirror disease's endpoint (e.g., elevated toxic metabolite, cell death, improper organelle assembly, …). For example, in Krabbe or Farber diseases, psychosine and ceramides respectively are toxic metabolites that can be quantified directly on patient primary skin fibroblasts and used as predictive readouts in phenotypic screenings. By the way, those are circulating metabolites used for diagnosis and eventually as part of the study of the natural history of the disease.

As few rare diseases are properly documented and very few models available, the patient biological material still remains one of the best models. Since the 1970s, skin fibroblasts have intensively been used to study inherited metabolic disorders (17). Indeed, these disorders result from anomalies affecting a single gene encoding a protein. Its deficiency often results in a cell-autonomous phenotype (18). Before the advent of genomics, skin biopsies were performed for diagnosis. Today, skin fibroblasts have become a very useful model for testing molecules and our choice to systematically look at impaired metabolic functions by mass spectrometry at high throughput. For instance, we used very long chain fatty acids and plasmalogens to study peroxisomopathies (19), or glycosaminoglycans in mucopolysaccharidoses (unpublished result). By adjusting the sample preparation process and the settings of the mass spectrometer, several phenotypic screenings were made possible in a couple of months and successfully led to the identification of potential drug candidates. The main strength of these metabolic screens lies in the choice of metabolites which in many cases turn out to be the clinical biomarkers of the disease.

In the context of genetic diseases, it is also necessary to consider the impact of the genotype on the effectiveness of identified treatments. The variety of mutations within the same gene, often explains a spectrum of symptoms and their level of severity. In line with their different clinical outcomes, these different mutations may not be equally rescuable by a single molecule. The risk is therefore to identify drugs effective only for a subset of patients, decreasing financial attractiveness for developers. It then justifies that a drug is only indicated for some mutations, as for ivacaftor (Kalydeco^®^) in cystic fibrosis. This is also the case in Zellweger spectrum disorders where the type of mutation directly impacts the type and severity of symptoms, and consequently the specificity of response to drugs (20). Adopting a strategy targeting focused patient populations sharing the same mutation and phenotype may then prove beneficial.

Whatever the size of the target population is, compassionate prescription of the existing drug, remains a good opportunity. For instance, ANSM (i.e., the health French authority for drugs) can frame off-label prescriptions provided that: (1) there is a therapeutic need and (2) the benefit/risk ratio of the medicine is presumed to be favorable, in particular based on scientific data on effectiveness and tolerance. The off-label access is accompanied by a patient monitoring protocol for collecting information regarding the effectiveness, adverse effects and actual conditions of use of the medication as well as, where applicable, the characteristics of the population eligible for the medicine. For ultra-rare disorders, where the population size is rarely in favor of running costly and complex clinical trials, compassionate prescription remains the best manner of generating first evidence of clinical safety and efficacy, supporting further studies toward a market authorization in the new indication. As there is a need of constant control by the health authorities, it will be interesting to extend the French initiatives for ultra-rare disease patients at the European level.

## Conclusion

In conclusion, the article emphasizes the largely untapped potential of drug repurposing as a viable and efficient strategy for developing treatments for ultra-rare disorders. Indeed, traditional drug development methods often overlook these diseases due to their rarity and the substantial costs involved. In that context, drug repurposing based on existing pharmacological agents and their known safety and PK profiles, can significantly reduce discovery time and costs, and fast translation to the clinic. The pioneering work of Apteeus, which combines phenotypic screening with drug repurposing, illustrates a holistic approach to identify new therapeutic applications for existing drugs. This method, particularly suited to inherited metabolic disorders, utilizes primary patient cells to accurately model those diseases and efficiently screen drugs library.

The integration of phenotypic screening in drug repurposing not only accelerates the identification of promising therapeutic candidates but also enhances our understanding of disease mechanisms. Despite challenges such as the need for robust disease models and the variability in patient responses due to different genetic mutations, the approach holds promise for delivering tailored treatments to those affected by ultra-rare conditions. Furthermore, compassionate prescription frameworks, as seen in France provide crucial pathways to link unbiased patient-based screening in the lab and initial clinical research, offering hope and potential therapeutic benefits.
